# Potentials and Challenges of Pervasive Sensing in the Intensive Care Unit

**DOI:** 10.3389/fdgth.2022.773387

**Published:** 2022-05-17

**Authors:** Anis Davoudi, Benjamin Shickel, Patrick James Tighe, Azra Bihorac, Parisa Rashidi

**Affiliations:** ^1^Department of Biomedical Engineering, University of Florida, Gainesville, FL, United States; ^2^Department of Medicine, University of Florida, Gainesville, FL, United States; ^3^Department of Anesthesiology, University of Florida, Gainesville, FL, United States; ^4^Department of Medicine, University of Florida, Gainesville, FL, United States

**Keywords:** intensive care unit (ICU), wearable device, computer vision, pervasive sensing, patient monitoring

## Abstract

Patients in critical care settings often require continuous and multifaceted monitoring. However, current clinical monitoring practices fail to capture important functional and behavioral indices such as mobility or agitation. Recent advances in non-invasive sensing technology, high throughput computing, and deep learning techniques are expected to transform the existing patient monitoring paradigm by enabling and streamlining granular and continuous monitoring of these crucial critical care measures. In this review, we highlight current approaches to pervasive sensing in critical care and identify limitations, future challenges, and opportunities in this emerging field.

## Current Critical Monitoring Paradigm

Critically ill patients in the intensive care unit (ICU) require constant monitoring. Currently, “continuous” monitoring of ICU patients is limited to automated vital sign measurements such as heart rate, blood pressure, body temperature, oxygen saturation, and respiratory rate. Other monitoring activities are limited by nurse availability for observing and documenting events, e.g., documenting falls or self-extubation events or detecting any exacerbation in important clinical indices such as mobility, agitation, pain, and consciousness. At present, assessment of these indices heavily relies on manual and repetitive examinations by nurses, leading to increased work pressure and the potential for burnout (1, 2). These manual indices also suffer from human error in data entry, observer subjectivity, and limited measurement granularity (3–5).

A more granular and continuous assessment of such critical care indices would enable a more comprehensive view of patient health. For example, granular functional status and behavioral assessment could lead to timely and personalized interventions based on data-driven guidelines. The need for continuous and automated monitoring of ICU patients has led researchers to incorporate non-traditional methodologies such as computer vision, wearable sensing technology, and various analytics algorithms (6). A comprehensive picture of the current state of the research in this domain will help outline the next steps and point out some of the questions that need to be answered. This work evaluates the feasibility of such monitoring approaches that are amenable to pervasive sensing in the ICU.

In the following sections, we detail current applications of pervasive sensing in ICU patient care settings. We then outline the knowledge gap in the literature, discuss current limitations, and highlight potential avenues for future research in augmenting traditional intensive patient care with pervasive sensing technology.

## Recent Advances in Critical Care Monitoring

Currently, monitoring ICU patients' functional status and behavioral aspects is limited in both granularity and information-richness. Pervasive sensing of the patient and their surrounding environment can provide a more comprehensive, continuous assessment of patient status. It can aid in quantifying patient health trajectories during the ICU stay. Two of the main avenues of research for pervasive sensing in the ICU involve using wearable accelerometers or computer vision devices, such as thermal or depth cameras, for monitoring the status of the patient and their environment. Wearable accelerometer devices, often resembling wristwatches, are lightweight, non-invasive, and easy to use. They allow for various computational analyses, do not pose any safety or comfort concerns for the patient, and do not impede the care procedures in the ICU. Wearable sensors have been previously used for quantifying human mobility and activity monitoring in various populations, and numerous analytic approaches showcase their potential uses for ICU patients (7–9). Applications of wearable accelerometers in the critical care setting are wide-ranging, including but not limited to physical activity (10–13) and energy expenditure monitoring (14), sleep detection (8, 15), agitation or sedation monitoring (16, 17), physiological signal monitoring (18, 19), fall detection (20, 21), delirium detection and subtyping (9, 22), sepsis subtyping (12), and frailty determination (23).

**Figure 1:**
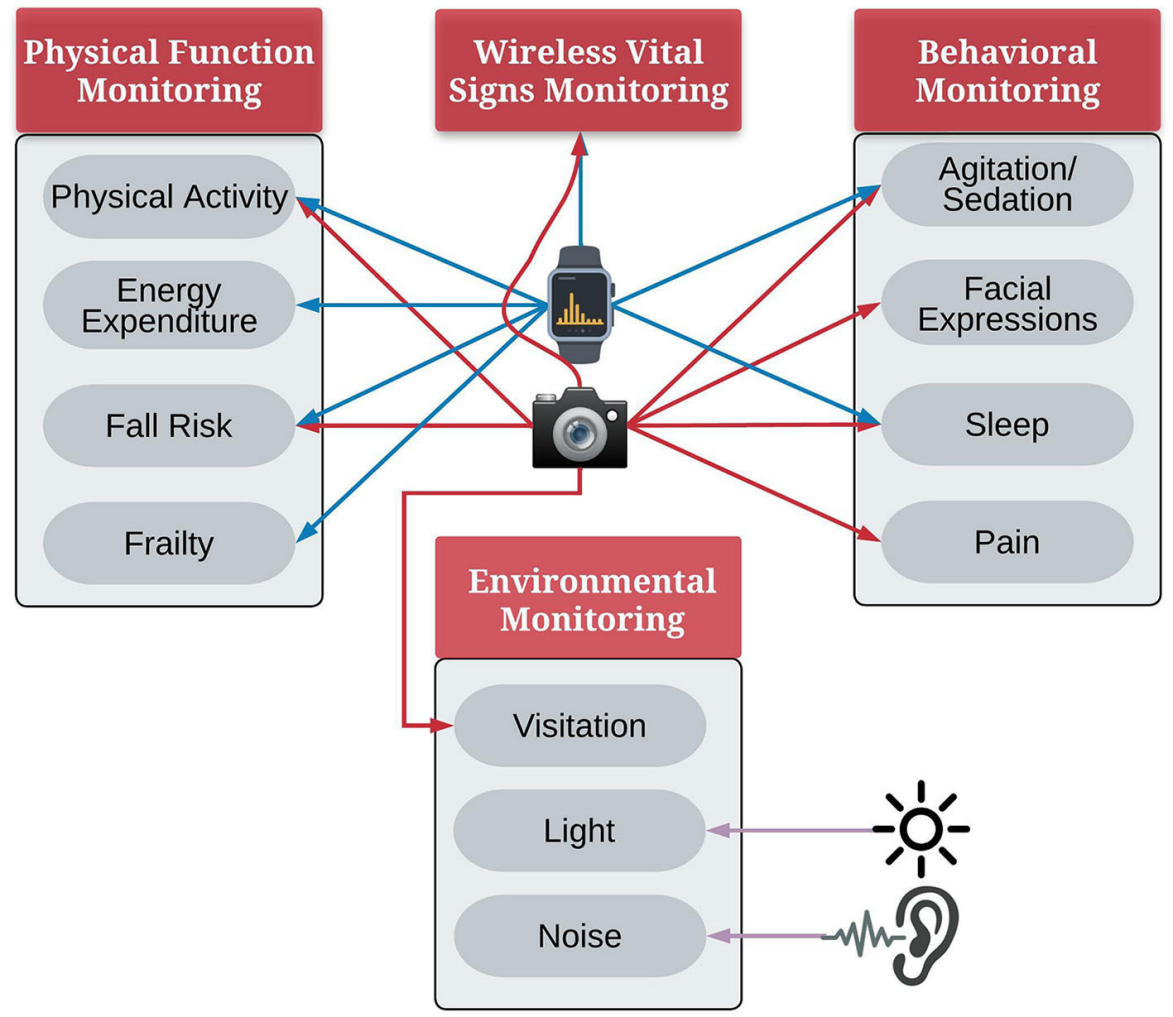
Applications of pervasive sensing for monitoring ICU patients, iconfinder.com.

Another potential avenue for pervasive ICU monitoring involves the application of computer vision techniques. Computer vision provides a means for non-contact sensing of the patient and their surroundings in the ICU, providing rich information on several physical functions and behavioral aspects. Possible applications of computer vision in the critical care setting fall into two categories: (a) healthcare team observation, such as measuring nursing workload (24) or monitoring hand hygiene (25), and (b) patient monitoring. This article focuses on the latter—computer vision applications for assessing patients and their environment. Previous research has demonstrated the potential applications of computer vision for fall detection (26, 27), sleep pose detection (28), agitation detection (29), physical activity monitoring (30, 31), head pose detection (22), physiological signal monitoring (32), and visitation detection (22, 33) in hospital settings (Figure 1).

## Physical Function Monitoring

ICU patients spend most of their time lying in bed, with significantly less time sitting in a chair, standing, or walking (22, 34). Patients' limited physical activity in the ICU has been linked with disruptions in circadian rhythm, a higher risk of delirium, and adverse outcomes in terms of cognitive and functional status at the time of hospital discharge and in the long term. Efforts at introducing physical therapy and early mobilization improve the patients' mobility and clinical outcomes such as delirium days, discharge disposition, and the risk of readmission or death (35–38). However, currently, there is a need for an objective, continuous, and accurate evaluation method to quantify the effect of rehabilitation practices. Additionally, such quantitative measures could be used to evaluate the association between a patient's activity levels and their outcomes during and after their ICU stay.

Existing clinical routine measurements of patients' mobility and physical status consist of limited standardized observational scores such as the ICU Mobility Scale (IMS) (39, 40). These observational evaluations aim to quantify patient mobility (40), but they still lack granularity and objectivity. Additionally, they provide limited information about mobility patterns' complex and dynamic nature throughout the ICU stay.

Computer vision and wearable accelerometer devices can provide more granular, objective, and continuous information on functional activity. Both approaches have been used to study physical activity in clinical settings to examine the association with health outcomes such as delirium or sepsis. However, there is limited research using computer vision or accelerometers in the critical care domain (Table 1), despite the high prevalence of sepsis or delirium in ICU patients. For example, delirium prevalence in specialized ICUs can be as high as 87%, and sepsis prevalence can be as high as 39% (46, 47).

**Table 1:** Peer-reviewed publications using wearable devices and computer vision in monitoring patients.

**Task**	**Data modality**	**References**
Physical activity	Vision Wearables	(10–12, 22, 28, 30, 31, 41–43)
Energy expenditure	Wearables	(14)
Fall risk	Vision Accelerometer	(20, 21, 26, 27, 44, 45)
Frailty	Accelerometer	(23)

### Physical Activity

Recent efforts in the field of computer vision have applied deep learning techniques to build models for automated physical activity and posture recognition. Different camera types have been used to detect patient mobility, including Red-Green-Blue (RGB) cameras, depth cameras, and cameras that capture both color and depth images, such as the Microsoft Kinect device. Multi-view settings using multiple cameras installed at different positions also have been used for capturing a more encompassing view of the patient room (28, 31, 41, 42).

Generally, automated detection of patient mobility using computer vision first requires patient identification in the scene. After patient recognition, manually annotated datasets are used to train the model to classify patient pose and mobility. Such systems have been able to accurately classify patients' high-level activities such as *nothing in bed (doing nothing, lying in bed), in-bed activity, out-of-bed activity*, and *walking* (30), and postures such as *lying in bed, sitting on the bed, sitting on the chair*, and *standing* (22) in the ICU. Depth camera-based systems have been able to classify four provider activities: “moving the patient into and out of bed” and “moving the patient into and out of a chair” without incorporating the challenging step of patient recognition (48, 49).

While computer vision techniques can identify a patient's posture, accelerometer devices can quantify the movement intensity. Wearable devices allow for convenient data collection and analysis since they provide continuous and patient-specific data streams. Wearable accelerometers have been used for examining physical activity patterns in different cohorts in various ICU settings, including delirium patients, sepsis patients, and patients with unilaterally motor impairment (10–13, 43, 50).

### Energy Expenditure

Building on activity intensity detection, wearable accelerometer devices have been used for determining energy expenditure. Previously energy expenditure estimation of accelerometer devices has been validated in the healthy adult population (14). However, it has been shown that energy expenditure is overestimated in ICU patients by comparing mechanical ventilation with indirect calorimetry. The current methodology of estimation of energy expenditure relies on the detection of physical activity. It does not incorporate physiological conditions such as fever with shivering that may alter the energy expenditure (51). Accurate estimation of energy expenditure in ICU patients enables optimizing enteral feeding details to prevent overfeeding and underfeeding, both of which increase the risk of infection and prolonged weaning from mechanical ventilation (52).

### Frailty

Accelerometer devices have also been used to detect frailty (23), a geriatric syndrome defined as “a clinically recognizable state of increased vulnerability, resulting from the aging-associated decline in reserve and function across multiple physiologic systems such that the ability to cope with every day or acute stressors is compromised” (53). There is increasing evidence of frailty being an indicator for decreased reserve and increased vulnerability in critical care patients (54, 55). Frailty has been shown to increase the risk of both adverse events such as death and discharge to skilled nursing homes and prolonged hospitalization and loss of independence after hospital discharge (56, 57).

### Falls

ICU patients experience decreased functional status and more muscle atrophy (58) exacerbated by minimal physical activity. They also suffer from impaired consciousness and attention (59), further compounded by disrupted circadian rhythm and sedation. While previous research has demonstrated the importance of physical activity in ICU patients, the increased risk of falls is brought on by confusion and agitation (60). Computer vision can detect falls in hospital settings (26, 27, 44, 45), as well as accelerometer-based monitoring systems (20, 21). Still, these approaches have not been adequately investigated in the ICU.

Despite the importance of accurate assessment and monitoring of physical activity, energy expenditure, fall risk, and frailty in ICU patients, few studies have investigated the use of pervasive sensing to facilitate assessment and monitoring.

## Behavioral Monitoring

### Facial Expressions

Behavioral indices such as pain facial expressions are different from physical activity indices. They do not elucidate gross variations in patients and thus could elude the nurses' observation. Facial expressions can also be influenced by sedative-hypnotics and analgesics commonly used in the ICU. While there is no validated facial expressions score in critical care, a few preliminary studies have examined anxiety-related facial expressions. Anxiety is highly prevalent in critical care patients (61, 62). However, it is rarely screened in routine care settings in the ICU (62). Computer vision approaches have been used for anxiety detection (63, 64), focusing on features such as head movement, mouth, and eye movement, and heart activity as indicators of anxiety. Patient head pose angle and variability have also been studied using computer vision and associated with pain and agitation indicators (22, 65–67).

### Pain

Continuous and objective monitoring of patient pain in the ICU, including for non-communicative patient populations, can pave the way for real-time adjustments to analgesics for optimal patient care, patient experience, and better health outcomes.

While wearable accelerometers have previously been used for studying pain (68), no study has investigated the relationship between pain and physical activity in the ICU settings, leaving unanswered the question of the complicated relationship between mobility, mental agitation, stress, and pain. The issue of pain in the ICU has many aspects. In addition to the potential effect of pain on a patient's physical activity, facial expressions and physiological signals may also be affected by pain. Previous work has investigated the feasibility of pain detection using vital signs (69, 70). Although this approach uses data routinely collected in the ICU, it has not shown strong specificity for pain detection. Formalizing facial expression of pain using facial action units (71) and advances in deep learning and computational power available have made it more plausible to move toward automated detection of facial expression of pain in the ICU. Facial expressions are assessed manually using several behavioral pain scales such as Non-verbal Pain Scale (NVP), Behavioral Pain Scale (BPS), and Critical Care Pain Observation Tool (CPOT), particularly for non-communicative patients (72–74). Researchers have used deep learning approaches to detect facial expressions of pain and to recognize individual facial action units associated with pain. Still, robust automated detection of pain in the ICU scene based on facial information requires more research and validation (75–79).

### Agitation/Sedation

Agitation is prevalent in the ICU and is a large factor in conditions such as delirium (80). Current methods for assessing delirium rely on transient rather than continuous assessment, which is an important limitation given the waxing and waning characteristics which help define delirium. Over-sedation has been shown to lengthen ICU duration and put a patient at higher risk for delirium (81). In comparison, under-sedation has been linked with increased agitation and a higher risk of self-extubation (82, 83). Optimizing sedation to better control patients' agitation may lower the patient's risk of removing endotracheal tubes (84). Similar to pain, accurate detection of a patient's agitation and sedation levels will improve the administration of sedative interventions to optimize clinical decisions. Previous research has used sensors for monitoring agitation in critical care settings. Agitation detection methods have shown strong performance using accelerometers (16, 17, 85), image-based approaches (29), and pupillometric video devices (86). Using wearable devices to study anxiety, researchers used Google Glass to discover that heart rate, but not spontaneous blink rate, changes in anxious patients (87).

### Sleep Detection

Previous studies have shown generally poor sleep quality in critical care settings (88). Sleep disruption in the ICU has been linked to various factors, including but not limited to the type and severity of the underlying medical condition, round the clock health care activities, enteral feeding, medication side effects, lack of natural light exposure and noise levels, and general disruptions to patients' circadian rhythm (88–90).

Sleep disturbance in ICU patients has been studied to determine its effect on patient outcomes and recovery (91) and has been shown to increase the risk of a longer stay in the ICU, worse discharge outcomes, impaired defense mechanism, and sleep disturbances that persist or develop after discharge (90). Determining a patient's sleep quality during their stay in the ICU allows for evaluating the effectiveness of the administered sleep hygiene interventions. Polysomnography, as the gold standard for studying sleep, has previously been investigated in ICU patients. However, polysomnography data require interpretation and might not be feasible for continuous data collection throughout the patient's stay in the ICU since it typically includes several EEG leads, electro-oculography, and chin electromyography (92, 93).

Accelerometer devices have been evaluated in quantifying sleep in healthy populations (15). However, previous studies have shown that they overestimate sleep in the ICU settings, possibly because the implemented sleep detection algorithms rely on a lack of physical activity in determining sleep events. ICU patients typically have low activity throughout the day, resulting in fractured sleep and shallower sleep stages (8). Other researchers have used computer vision to detect sleep pose, but these studies have been investigated based on data from healthy adults, limiting the generalizability of their performance to the ICU setting (28, 94).

## Environmental Monitoring

ICU patients spend most of their ICU stay in one room, leaving the room only for medical procedures. Information about the ICU room environment can enhance our understanding of possible contributing factors to patients' recovery speed. Wearable accelerometer devices are more suited for monitoring patients' physical activity, but they do not capture any information about patients' surroundings. Computer vision techniques offer additional opportunities for capturing and studying the effects of environmental factors on patients' recovery trajectories.

### Visitation Detection

To encourage sleep hygiene, hospitals generally implement official guidelines for regulating the presence of visitors in the ICU. However, visitations and interactions with the environment may be beneficial in improving patients' experience by reducing their anxiety, leading to a lowered risk of delirium and an overall more positive experience during their ICU stay (95, 96). Accurate detection of the number of visitors and healthcare personnel in the room and environmental factors such as a room's noise and light at all hours allows for quantifying the effects of such disruptions on patients' sleep quality and circadian rhythm integrity. Computer vision has been used to determine the number of people in ICU care rooms (31, 33, 97) to understand the association between visitation and clinical care disruptions to patients with patients' sleep hygiene and outcomes. Such information could assist in developing more accurate evidence-based visitation and sleep quality guidelines for ICU patients.

### Light and Noise Monitoring

Light and noise intensity levels are primary contributors to sleep deprivation and fragmentation in the ICU (91). Previous studies have demonstrated the feasibility of using affordable light and noise intensity sensors in ICU rooms (22, 98). Several studies using noise intensity sensors in the ICU have determined that noise level frequently surpasses the levels recommended by the World Health Organization (WHO) (99, 100). Various sleep hygiene interventions in the ICU population incorporate light and noise exposure limits with mixed results in efficacy. Accurate, continuous light and noise intensity measures can enhance our understanding of the effect of environmental factors on patient sleep and the efficacy of sleep hygiene guidelines (90).

## Non-Contact Vital Sign Monitoring

In addition to novel physical activity and behavioral indices, the use of computer vision and wearable devices has enabled non-contact monitoring of vital signs such as heart rate and blood pressure in the ICU. Such devices could remove the need for electrodes and cuff-based devices sensitive to movement artifacts, prone to detachment, and restricting patient movement. Recent research in computer vision and wearable devices has focused on the feasibility of non-contact monitoring of vital signs, including the use of RGB and thermal cameras for contactless estimation of heart rate, blood pressure, blood oxygen saturation, and respiration rate in research, hospital and ICU settings [e.g., (32, 101–106)]. Wearable devices have been used for measuring heart rate, oxygen saturation, and blood pressure in hospital and ICU patients [e.g., (18, 19, 107)].

## Post-Discharge Monitoring

Critical care patients often require longitudinal monitoring and follow-up visits after discharge from the hospital. Patients' discharge destination can vary depending on the health status, ranging from home and home care for more stable patients to hospice for those who need more care with a lower chance for recovery. While clinicians have access to many tools to assess patients and determine a prognosis, current assessments do not extend well to post-discharge monitoring, resulting in less quantifiable information about post-discharge recovery (108). Computer vision approaches are not suitable in these scenarios due to technical and especially privacy concerns. However, wearable sensors can be used for physical activity monitoring and patient recovery (109), facilitating more comprehensive evaluations between follow-up visits. There is currently limited research on using this methodology to monitor the improvement in patients' functional status in free-living settings among survivors of critical illness (109–111). With the increasing popularity of accelerometer-equipped smartwatches, it is also possible to monitor physical activity before and after a patient's ICU stay to examine its relation with health recovery.

## Technical Challenges and Future Directions

**Figure 2:**
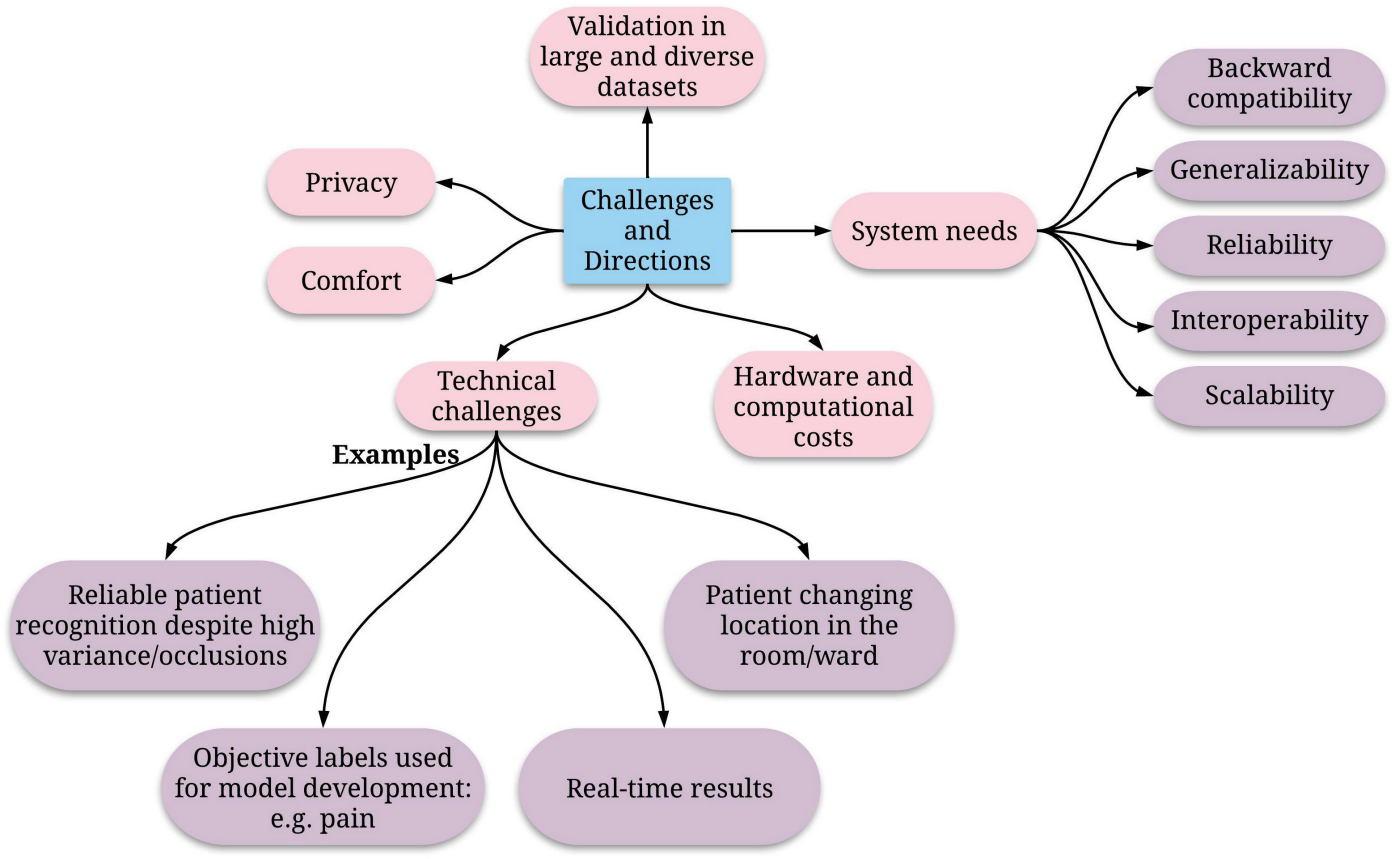
Directions of future research to enable effective and reliable pervasive monitoring in the ICU.

While the current body of literature shows strong potential for using these novel approaches in critical care for faster, more personalized, and more accurate clinical decisions, several challenges need to be addressed (Figure 2).

### Ethical and Privacy Concerns

Deep learning methods discussed in many of the computer vision-based studies mentioned in this review require large, labeled datasets for training and validation. The developed models need to be validated in diverse populations in ICU settings and consider age, gender, primary diagnosis, and race. However, ethical and legal reasons rightly prevent the construction of public datasets to protect the privacy of patients, their visitors, and the healthcare team (112). Existing Privacy guidelines typically mandate deidentification and temporary recordings, while the current state of research still requires raw recordings of the ICU room for post-event annotations and analysis. While research in this direction could potentially facilitate the clinical workflow, researchers should be cognizant of the privacy implications and the tradeoff between privacy and technological benefits.

### Computer Vision

An essential intermediate step in several computer vision approaches is detecting a patient in the room of each video frame. Previous research (113–115) has shown promising results in facial recognition in each single frame or tracking the patient face during the recording, but most of these studies are validated on an ideal frontal full-face view of a patient's face or with standard lighting. Real-world ICU rooms may be crowded, with varying degrees of lighting and numerous objects that could partially obscure the patient's face, such as ventilators or oxygen masks. Patient face identification will be even more challenging due to variations in the face angle and obscuring elements such as facial hair or glasses. Improved patient recognition adapted to the ICU setting will make computer vision solutions more robust. Because of the variable location of the bed and patient, the ideal developed methodology should be agnostic to the bed/patient location and position.

### Sleep Detection

Sleep detection methods still require further research for reliable use in ICU patients. Future approaches may focus on multimodal models and using wearable sensors to collect information on activity, heart rate, and body temperature. Current wearable activity sensors determine sleep based on a lack of activity and wake periods, but this does not account for the minimal activity of the ICU patients, thus resulting in low specificity.

### Pain Assessment

Researchers also need to consider the effect of pain relief medications, nociceptive generators, and interindividual differences in pain processing in studying patients' pain. However, this is a challenging concept since medication effect changes over time depending on pharmacokinetics such as age, sex, weight, body surface area, renal and hepatic function, fluid shifts, medication dosage, time since administration, administration route, and drug-drug interactions. This complexity often increases for continuous infusions of medications. Additionally, model development relies on patient self-report and nurses' observation for non-verbal patients. The uncertainty regarding how to translate pain intensity assessments into objective, rational clinical decisions for analgesic therapies that reduce pain intensity and patient risk further complicates developing generalizable and reliable models. There is also a need to integrate outcomes pertaining to pain, analgesic requirement, and “patient function” to provide a more holistic perspective on recovery trajectories.

### Wearable Devices

Another implementation challenge for using wearable devices in the ICU is the requirement of current devices to be tightly secured on the skin to better capture patients' subtle movements. This may prove to be an inconvenience for some patients, as continuous contact with the skin might cause irritation or even could pose risks for infection, tissue ischemia, compartment syndrome, and wound breakdown. Furthermore, for patients with medical equipment on their wrists, wrist-worn accelerometer devices might not be an option unless future medical equipment includes built-in accelerometers.

### Forward-Compatibility

The inclusion of multiple data streams for different patient care tasks using pervasive sensing also requires *generalizability, interoperability, scalability*, and *reliability* of the systems. *Interoperable* and *generalizable* systems can operate together and be incorporated into different monitoring platforms, while *scalable* systems will accommodate collecting data from a larger number of patients. The accompanying analytical algorithms need to be *adaptable* for new hardware choices. Moreover, there must be a consensus on the measured variables better to evaluate the performance of proposed methods and devices. Ultimately, the positive impact of such systems in critical care needs to be rigorously assessed and validated.

### Model Validation With Minimal Data Burden

To improve the adoption of pervasive sensing in routine care in the ICU, developed models and devices should be easily manageable by the care team with minimal or no required training. Any developed model should be validated to reduce the false alarm rate in the ICU -as is investigated with vital sign-based alarms (116)-and should optimize the visualization approach to prevent data fatigue. The presentation of new information should be determined by considering the preferences of the physicians and their team and could include facets such as a daily summary, continuous display or separate tabs, or simple alarms for specific, pre-determined events.

### Real-Time Models

Ultimately, any developed detection and prediction model should report a patient's status in real-time to allow the ICU team to implement timely interventions, such as incorporating more active physical therapy regimens, improving sleep hygiene routines, and adapting administered medications. The necessary communications infrastructure and reporting medium should be optimized to avoid alarm fatigue to the already overburdened ICU nurses.

## Conclusion

Patients in the ICU have diverse and heterogeneous health backgrounds, which necessitate more personalized and dynamic treatments and interventions. This calls for developing monitoring methodologies that provide continuous, objective, and quantifiable patient information. Traditional monitoring of vital signs, nursing observations, and self-reported pain scores is essential but does not provide a comprehensive view of the patient's overall health status.

Advances in computation and computer vision fields and the development of accurate measuring devices such as wearable accelerometers have introduced more options for patient monitoring in-home, community, and hospital settings. The acute nature of health events in the ICU can benefit from pervasive, passive sensing methodologies that reduce nurses' workload and replace some of the tasks that require repetition of measurements, such as detection of pain and agitation. Moreover, pervasive sensing technology can enable measuring indices that were not previously recorded, including a patient's physical activity level, facial expressions, and head pose variations. Classification algorithms trained on the data from similar scenarios may allow for more timely prediction of adverse events such as falls and delirium, enabling the healthcare team to prevent their occurrence.

Many of the proposed domains of the ABCDEF bundle,^1^ an evidence-based guide for clinicians to improve ICU patients' recovery and outcomes that emphasize pain assessment, prevention, and management (117), require accurate monitoring of the patients during their stay in the ICU. Continuous monitoring of pain levels using computer vision approaches will be helpful for continuous and accurate pain assessment and ultimately for real-time adaptation of pain medications. The choice of analgesia and sedation is another domain in this guideline that can benefit from continuous monitoring of the patients' sedation levels to personalize analgesia choice. Quantifying patients' mobility also improves the evaluation of the effectiveness of the administered mobility and exercise regimens. Moreover, the use of accelerometer and vision sensors to detect delirium can improve delirium assessments, as proposed in the ABCDEF bundle.

## Author Contributions

AD and PR developed the concept for the study. AD drafted the manuscript and designed and created the figures. PR substantially revised the manuscript and revised the figures. BS, AB, and PT substantially revised the manuscript. All authors were approved the final manuscript.

## Funding

PR was supported by 5R01AG055337 from National Institute of Aging (NIH/NIA), CAREER award 1750192 from the National Science Foundation, 1R01EB029699 and 1R21EB027344 from the National Institute of Biomedical Imaging and Bioengineering (NIH/NIBIB), R01GM-110240 from the National Institute of General Medical Science (NIH/NIGMS), 1R01NS120924 from the National Institute of Neurological Disorders and Stroke (NIH/NINDS), and R01 DK121730 from the National Institute of Diabetes and Digestive and Kidney Diseases (NIH/NIDDK). AB was supported R01 GM110240 from the National Institute of General Medical Sciences (NIH/NIGMS), R01 EB029699 and R21 EB027344 from the National Institute of Biomedical Imaging and Bioengineering (NIH/NIBIB), R01 NS120924 from the National Institute of Neurological Disorders and Stroke (NIH/NINDS), and by R01 DK121730 from the National Institute of Diabetes and Digestive and Kidney Diseases (NIH/NIDDK). PT was supported by 5R01GM114290 and 5R01AG055337.

## Conflict of Interest

The authors declare that the research was conducted in the absence of any commercial or financial relationships that could be construed as a potential conflict of interest.

## Publisher's Note

All claims expressed in this article are solely those of the authors and do not necessarily represent those of their affiliated organizations, or those of the publisher, the editors and the reviewers. Any product that may be evaluated in this article, or claim that may be made by its manufacturer, is not guaranteed or endorsed by the publisher.

## Footnotes

^1^**A**ssess, Prevent, and Manage Pain, **B**oth Spontaneous Awakening Trials (SAT) and Spontaneous Breathing Trials (SBT), **C**hoice of analgesia and sedation, **D**elirium: Assess, Prevent, and Manage, Early mobility and **E**xercise, and **F**amily engagement and empowerment.
